# Reciprocal Interactions between Cadmium-Induced Cell Wall Responses and Oxidative Stress in Plants

**DOI:** 10.3389/fpls.2017.01867

**Published:** 2017-10-31

**Authors:** Christophe Loix, Michiel Huybrechts, Jaco Vangronsveld, Marijke Gielen, Els Keunen, Ann Cuypers

**Affiliations:** Environmental Biology, Centre for Environmental Sciences, Hasselt University, Diepenbeek, Belgium

**Keywords:** cadmium, oxidative stress, cell wall, pectin, lignin, ascorbate, hydrogen peroxide

## Abstract

Cadmium (Cd) pollution renders many soils across the world unsuited or unsafe for food- or feed-orientated agriculture. The main mechanism of Cd phytotoxicity is the induction of oxidative stress, amongst others through the depletion of glutathione. Oxidative stress can damage lipids, proteins, and nucleic acids, leading to growth inhibition or even cell death. The plant cell has a variety of tools to defend itself against Cd stress. First and foremost, cell walls might prevent Cd from entering and damaging the protoplast. Both the primary and secondary cell wall have an array of defensive mechanisms that can be adapted to cope with Cd. Pectin, which contains most of the negative charges within the primary cell wall, can sequester Cd very effectively. In the secondary cell wall, lignification can serve to immobilize Cd and create a tougher barrier for entry. Changes in cell wall composition are, however, dependent on nutrients and conversely might affect their uptake. Additionally, the role of ascorbate (AsA) as most important apoplastic antioxidant is of considerable interest, due to the fact that oxidative stress is a major mechanism underlying Cd toxicity, and that AsA biosynthesis shares several links with cell wall construction. In this review, modifications of the plant cell wall in response to Cd exposure are discussed. Focus lies on pectin in the primary cell wall, lignification in the secondary cell wall and the importance of AsA in the apoplast. Regarding lignification, we attempt to answer the question whether increased lignification is merely a consequence of Cd toxicity, or rather an elicited defense response. We propose a model for lignification as defense response, with a central role for hydrogen peroxide as substrate and signaling molecule.

## Introduction

During their evolution, plants have acquired certain mechanisms to cope with various environmental stresses such as drought, salinity, and temperature stress. Considering that our climate is changing at a rapid pace, these external pressures are becoming even more pronounced. Furthermore, anthropogenic activities such as mining, the use of phosphate fertilizers, burning fossil fuels, and metal-related industrial processes have caused an increasing release of metals in the environment (Gallego et al., 2012). Some of these elements are of fundamental biological importance to both humans and plants. For example, iron (Fe) is essential for various plant processes, including nitrogen assimilation, photosynthesis and hormone biosynthesis. Heme proteins containing a redox-active Fe atom such as catalase (CAT), class I and class III peroxidases (PODs), and NADPH oxidase have a pivotal function in both the production and scavenging of reactive oxygen species (ROS) (Tchounwou et al., 2012). Other metallic micronutrients such as copper (Cu), manganese (Mn), molybdenum, nickel, and zinc (Zn) are essential for all higher plants (Hänsch and Mendel, 2009). On the other hand, some metals are phytotoxic even when they are taken up in low amounts. Due to its bioavailability and detrimental effects on human health, cadmium (Cd) is one of the most studied toxic metals (Clemens, 2006; Nawrot et al., 2010). As plants constitute an important link between the soil elemental composition and the food chain, it is highly important to investigate how plants deal with Cd exposure from uptake to potential acclimation.

Although Cd is not redox-active, it has the potential to increase the production of ROS via indirect mechanisms such as the replacement of redox-active metals in proteins and activation of NADPH oxidases (Cuypers et al., 2010). This increase in reactive radicals has the capability of tipping the cellular redox balance in favor of the pro-oxidants, which can result in oxidative damage to proteins, DNA, and membrane lipids. Therefore, a cellular antioxidant defense system is mounted to restore this disturbed redox balance. These defense mechanisms consist of enzymes such as superoxide dismutase (SOD), CAT and ascorbate peroxidase (APX) and metabolites such as glutathione (GSH) and ascorbate (AsA). In addition, GSH serves as a precursor for phytochelatins (PCs). These molecules have the capacity to chelate Cd, thereby reducing its toxicity (Jozefczak et al., 2015; Parrotta et al., 2015). This intracellular defense system only has the ability to deal with negative effects of Cd to a certain extent. In this regard, the plant cell wall is an important plant component as it represent the first contact point of plants with Cd, and has an important function in keeping excess Cd out of the cell. However, the number of studies focusing on cell wall-related responses to Cd exposure is scarce (Parrotta et al., 2015). The cell wall is often considered as a rigid, unchanging structure, but it is currently proven to be a dynamic construction with exceptional strength, while its flexibility is still preserved (Banasiak, 2014). The role of the cell wall in plant responses to trace metal exposure was reviewed by Krzesłowska (2011). They concluded that the capacity of the cell wall to retain trace metals mainly depends on the number of negatively charged groups present within the cell wall, and that these groups are mainly found in low-methylesterified pectins (Krzesłowska, 2011). Furthermore, Parrotta et al. (2015) recently discussed the dual property of plant cell walls being a barrier or target for metals, taking Cd as an example. They focused on different cell wall compositions in early- and later-diverging embryophytes as well as differences between plant organs and tissues and how this variability might be important in regard to Cd accumulation taking also hyperaccumulators into account.

In the current review, the focus on cell wall-related responses in Cd-exposed plants is (1) on the induction of primary cell wall remodeling and its relation to the uptake of essential plant nutrients and (2) on the activation of lignin biosynthesis in relation to Cd-induced oxidative stress. For the latter, an important role is attributed to AsA as it functions as a major antioxidant, but also shares biosynthesis components with cell wall precursors. In addition, both AsA and Class III lignifying POD enzymes participate within a complex redox network, in which hydrogen peroxide (H_2_O_2_) is put forward as a major sensor of the redox balance, functioning both as a signaling molecule to induce lignin biosynthesis and as a substrate for lignifying PODs.

## Cadmium Impairs Proper Nutrient Homeostasis

Plants exposed to Cd have been shown to display excessive root damage, inhibition of the photosynthesis system, alterations of various metabolic enzymes, and a reduced biomass production. These physiological disorders might be an indirect effect caused by an impairment of mineral nutrition within the plant due to Cd exposure (Hédiji et al., 2015). Therefore, in this section, a short overview is given on (1) how Cd enters the plant system and (2) which effects Cd uptake has on the uptake of essential elements and how cell wall remodeling plays a role in mediating a disturbed nutrient homeostasis.

### Cadmium Uptake by Plants

Cadmium is a non-essential element that is easily taken up by plants, although no distinct Cd transporters are present (Li et al., 2017). As described by Wagner (1993), free Cd ions are opportunistic hitchhikers, entering the root system through mechanisms specifically designed for essential nutrients such as Fe, Zn, and calcium (Ca) (Clemens, 2006). The best-studied transporters include members of the ZIP (zinc-regulated transporters, iron-regulated transporter-like protein) and the Nramp (natural resistance-associated macrophage protein) families. The *Arabidopsis* iron-regulated transporter 1 (IRT1) protein is an important metal transporter of the ZIP family (Connolly et al., 2002). Depletion of Fe results in upregulation of Fe uptake systems, subsequently leading to a higher Cd influx as well (Wagner, 1993). Members of the Nramp transporters are mostly studied in yeast and their role in plants are far from fully understood. Most research is conducted on O*ryza sativa*, since it is both a model organism and a major source of Cd within the human diet (Clemens and Ma, 2016). There are seven members of the OsNramp family, however, the function of OsNramp2, OsNramp6, and OsNramp7 is still unknown (Wu et al., 2016). It was shown that OsNramp5 is a major transporter of both Mn and Cd into the interior of root cells (Sasaki et al., 2012). OsNramp1 could transport Fe and Cd in yeast and is proposed to be involved in Cd accumulation in plants as well (Takahashi et al., 2011). Overexpression of *AtNramp6*, which encodes for an intracellularly localized transporter, led to Cd hypersensitivity (Cailliatte et al., 2009). Studies including metal hyperaccumulating plants provide useful insights into the more general mechanism of metal absorption (Verbruggen et al., 2009; Clemens et al., 2013). Even under normal Zn exposure, ZIP transporters were expressed at a much higher level in the Zn hyperaccumulators *Arabidopsis halleri* and *Noccaea caerulescens* (previously known as *Thlaspi caerulescens*) (Krämer, 2010). In *N. caerulescens*, ZNT1 transporters mediate high-affinity Zn and low-affinity Cd uptake (Pence et al., 2000). Finally, it was shown that Cd is able to permeate *Arabidopsis thaliana* guard cells through Ca channels, thereby disrupting proper Ca signaling (Perfus-Barbeoch et al., 2002). In summary, Cd takes advantage of transport systems for essential elements such as Ca, Fe, and Zn to enter the plant.

### Cadmium Affects Plant Nutrient Uptake

Similarities in physicochemical properties between Cd and other divalent cations, e.g., Ca, Zn and magnesium (Mg), may antagonize the uptake of the latter (Kabata-Pendias and Pendias, 2001; Safarzadeh et al., 2013; Cannata et al., 2014). However, the opposite process in which nutrients are taken up more easily by plants under Cd exposure, can also occur (Kabata-Pendias and Pendias, 2001; Bertoli et al., 2012). It is obvious that Cd-induced responses on the uptake of plant nutrients are not straightforward and can become even more complex, following a positive or negative quadratic curve with increasing amounts of Cd (Bertoli et al., 2012; Cannata et al., 2014). Therefore, one must be careful drawing general conclusions and needs to take the experimental design and plant species into consideration. First of all, nutrient content is differentially affected in distinct plant organs (Wu et al., 2005; Douchiche et al., 2012). The root system is the first structure to come into contact with Cd. Since its major function is to translocate soil nutrients and water to the aboveground parts, this process is often disturbed in Cd-exposed plants. Secondly, plant species not only vary from each other in terms of their tolerance to Cd, they also react differently when examining nutrient concentrations after Cd exposure (Liu et al., 2003; Bertoli et al., 2012; Çikili et al., 2016). For example, 20 different varieties of *O. sativa* cultivars showed highly different responses related to the absorption and translocation of essential elements when exposed to Cd (Liu et al., 2003). Within this study, positive correlations were found between Cd and Fe, Zn and Cu. The results for Mn were only significant in the roots and no correlation was found for Mg in both roots and shoots of Cd-exposed plants. Thirdly, contrasting results can be explained by the use of different experimental systems in which the bioavailability and speciation of Cd can strongly differ, e.g., between hydroponic and soil-based setups (Azevedo et al., 2005b,c; Gonçalves et al., 2009; Dupae et al., 2014). Other important growth parameters such as light intensity, temperature, and humidity are usually specified by authors, yet knowledge in regard to how these factors interact with Cd exposure and downstream responses seems to be lacking (Dupae et al., 2014). Finally, also the developmental stage and aging of plants greatly influences the nutrient concentrations in different plant organs and should therefore be taken into account (Liu et al., 2003; Anjum et al., 2008; Januškaitienė, 2012; Januškaitienė and Dikšaitytė, 2014). For example, applying fertilization was able to alleviate the toxic effects of Cd on the photosynthetic system of *Pisum sativum* only at the stage of leaf development, but not at the stage of lateral shoot development (Januškaitienė and Dikšaitytė, 2014).

Up till now, only a few studies have investigated the underlying mechanisms by which Cd could affect the uptake and translocation of essential elements. One possible mechanism underlying these disturbances is cell wall remodeling. In a study using *A. thaliana*, leaf chlorosis was observed after Cd exposure mainly as a result of Cd-induced Fe deficiency in the shoots (Xu et al., 2015). Upon Cd exposure, the pectin and hemicellulose content of the root cell walls increased, leading to a major retention of Fe in the roots and hence a decrease of the Fe concentration in the shoots. In addition to Fe, Zn, and Cu translocation were also significantly reduced. In another study with *A. thaliana*, either phosphorus deficiency or Cd exposure resulted in similar effects such as growth retardation and leaf chlorosis (Zhu et al., 2012). However, when both conditions were combined, the chlorosis effect was partially reduced and chlorophyll content was significantly higher. Under these circumstances, a lowered pectin and hemicellulose1 content in the root cell walls were observed as compared to the Cd exposure condition alone. This led to both a lowered Cd retention in the roots and less Cd accumulation in the shoots, however, no information on Fe concentration was provided. These results emphasize the need for research on cell wall remodeling upon Cd exposure with special attention to alterations in nutrient concentrations.

## Cadmium Induces Cell Wall Remodeling

**FIGURE 1:**
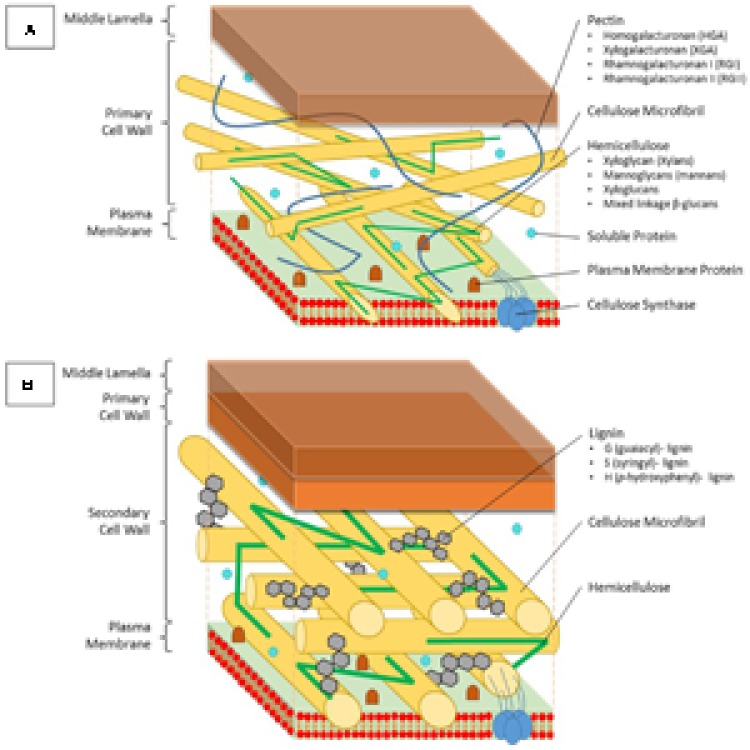
Structure and composition of the primary and secondary cell wall of plants. **(A)** The primary cell wall is located outside of the plasma membrane and consists of cellulose microfibrils, which are constructed by cellulose synthase complexes, hemicellulose, lignin and soluble proteins. Hemicellulose binds to the surface of the cellulose microfibrils and can be divided into four groups; xyloglycan (xylans), mannoglycans (mannans), xyloglucans, and mixed linkage β-glucans. Pectins form a hydrated gel between the cellulose-hemicellulose network and consists of four pectin domains: homogalacturonan (HGA), xylogalacturonan (XGA), rhamnogalacturonan I (RGI), and rhamnogalacturonan II (RGII). **(B)** The secondary cell wall is constructed between the primary cell wall and the plasma membrane. Between the typically more arranged cellulose microfibrils, lignin molecules are impregnated, thereby replacing pectin molecules. Lignin is a complex phenolic polymer consisting of three monolignol subunits: G (guaiacyl)-, S (syringyl)- and H (*p*-hydroxyphenyl)-lignin.

On the onset of this section, an overview is given on the general components that constitute the primary cell wall (**Figure 1A**), as well as the formation of a rigid secondary cell wall by the incorporation of lignin (**Figure 1B**). Subsequently, the importance of the cell wall as an effective barrier against Cd uptake is illustrated. Finally, primary and secondary cell wall modifications, as plant responses to Cd exposure, are focused upon in more detail.

### The Structure of the Plant Cell Wall

The main building blocks of the primary cell wall are proteins and polysaccharides such as cellulose, hemicellulose, and pectin (**Figure 1A**). Formation of a secondary cell wall is accomplished by adding phenolic components such as lignin in between the polysaccharide molecules (**Figure 1B**). Cellulose is the most abundant organic compound on earth (Klemm et al., 2005). Glucan chains consisting of D-glucose molecules cluster into cellulose microfibrils, which are embedded into a polysaccharide matrix. This biosynthesis process is accomplished by cellulose synthase localized in the plasma membrane using an UDP-glucose donor. Once these microfibrils are formed, they are tightly interconnected via hemicellulose, while pectin crosslinks the polymers (Gibson, 2012). Hemicellulose can generally be segregated into four groups based on its structure: xyloglycan, mannoglycans (mannans), xyloglucans, and mixed linkage β-glucans (Ebringerová, 2006). The amount of each structure present in the cell wall might vary depending on the plant species. Because of major differences in composition, the primary cell wall can be divided into two subtypes (Vogel, 2008). Pectin molecules make up 30% of the primary cell walls, yet they contain up to 70% of all negatively charged groups (Jarvis, 1984). They fixate the cellulose microfibrils and other polysaccharides by covalent and non-covalent binding, resulting in the formation of a hydrated gel phase. Pectins consist of four polysaccharide domains: homogalacturonan (HGA), xylogalacturonan (XGA), rhamnogalacturonan I (RGI), and rhamnogalacturonan II (RGII). The first domain, also being the most important in binding both divalent and trivalent metals, is synthesized within the Golgi apparatus and transported to the cell wall in a highly methylesterified form (Krzesłowska, 2011). Here, the newly formed pectin gets partially demethylated, which allows it to interact with other pectin fibers. Two free carbonyl groups can bind with each other using a Ca ion, thereby forming an egg-box structure (Grant et al., 1973). Trivalent aluminum as well as divalent ions such as Cu, Zn, Cd, and lead (Pb) are capable of replacing Ca within this structure. Two RGII domains are also able to create a dimer through the formation of boron diester bonds, thereby creating a more complex pectin structure (Yoneda et al., 2010). Furthermore the degree of methylated pectin can further be adjusted by a pectin methylesterase (PME), which creates low-methylesterified pectin by esterification of a more esterified pectin form.

**FIGURE 2:**
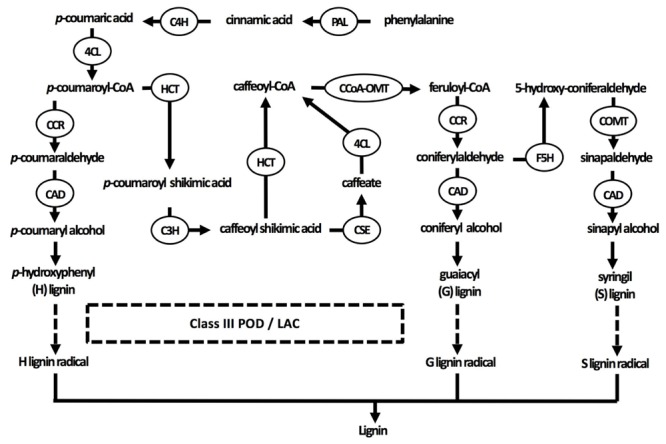
Current understanding of the lignin biosynthesis pathway in plants. Three specific monolignol subunits are synthesized from phenylalanine by enzymes that are sometimes able to catalyze different reactions in the pathways of all three monolignols. Monolignols are subsequently transported to the cell wall, where they are radicalized by class III peroxidases and laccases in the final step of lignification (indicated by dashed arrows). 4CL, 4-coumarate-CoA ligase; C3H, coumarate 3-hydroxylase; C4H, cinnamate 4-hydroxylase; CAD, cinnamyl alcohol dehydrogenase; CCoA-OMT, caffeoyl-CoA *O*-methyltransferase; CCR, cinnamoyl-CoA reductase; COMT, caffeic acid *O*-methyltransferase; F5H, ferulate 5-hydroxylase; HCT, *p*-hydroxycinnamoyl-CoA: quinate/shikimate *p*-hydroxycinnamoyltransferase; LAC, laccase; PAL, phenylalanine ammonia-lyase; POD, peroxidase.

Secondary cell walls are deposited in specialized tissues such as xylem and sclerenchyma, providing strength, which allows plants to grow upright, and transport water efficiently (Wang et al., 2013; Zhong and Ye, 2015). Secondary cell walls are primarily made up of three polymers: cellulose, hemicellulose, and lignin (**Figure 1B**). The proportion of these polymers varies widely between plant species and tissues, ranging from over 20% of lignin in certain hardwoods to none in phloem fibers of *Cannabis sativa* or *Linum usitatissimum* (Zhong and Ye, 2015). In the context of this review, the focus of the secondary cell wall in relation to Cd lies on lignin due to its close relation to redox systems as will be elaborated upon later. Lignin originates from the oxidative polymerization of monolignol subunits. Its biosynthesis starts with the conversion of the amino acid phenylalanine to cinnamic acid by the key enzyme phenylalanine ammonia lyase (PAL) and subsequently to *p*-coumaric acid (**Figure 2**). After conversion of *p*-coumaric acid to *p*-coumaroyl-CoA by 4-coumarate-CoA ligase, *p*-coumaroyl-CoA can follow one of three pathways that are specific but still share common enzymes, leading to the formation of three possible alcohols. These alcohols are further converted to specific monolignol subunits: guaiacyl (G), *p*-hydroxyphenyl (H), and syringyl (S) lignin. The last step of monolignol synthesis is not fully elucidated yet. In angiosperms, however, it was found that the final conversion step from sinapyl alcohol to S lignin is catalyzed by sinapyl alcohol dehydrogenase (Li et al., 2001). Once formed in the cytosol, an unknown mechanism transports monolignols to the cell wall (Boerjan et al., 2003). In the apoplast, laccases (LACs) and PODs, which are discussed in section “Cadmium Stimulates POD Activity Leading to Increased Lignification,” radicalize monolignols, which then polymerize into lignin (**Figure 2**) (Vanholme et al., 2010). Additionally, specialized paracellular structures form an exclusion barrier in the root endodermis. This structure is called the Casparian strip. Although it was thought to be made up of lignin and suberin, it was shown to consist exclusively of lignin in *A. thaliana* (Naseer et al., 2012). The Casparian strip is a major factor in limiting Cd uptake by roots (Seregin et al., 2004; Shi et al., 2016). Casparian strips in *Salix caprea* originating from polluted soils grew closer to the root tip as an adaptive response to Cd exposure. However, this did not result in a lower Cd accumulation in the aboveground plant parts as compared to *Salix* plants originating from unpolluted soils (Vaculík et al., 2012). This was due to *Salix* plants from non-polluted soils developing apoplastic barriers earlier when exposed to Cd and an apparent delay thereof in *Salix* originating from polluted soils. The latter mechanism remains poorly understood. Additionally, Cd might be translocated in young roots where Casparian strips are not fully formed yet (Hardiman and Jacoby, 1984). A role for the Casparian strip in Cd uptake is apparent, opening the window for more research to increase our understanding on how it specifically affects root Cd uptake and translocation.

To optimize plant yield on marginal soils, lignification is a crucial process to understand, as it is one of the key factors determining the economic success of a harvest in many biotechnological applications. In particular, it is an important factor in the application of plants in the non-food or non-feed bioindustry at which is aimed to perform agriculture on polluted soils. The general undesirability of lignin in crops for bioethanol production or conversely its positive effects on the burning properties of wood pellets are only two examples of a myriad (Brebu and Vasile, 2010; Stevens and Gardner, 2010).

### The Cell Wall as the Most Important Structure Retaining Cd

The cell walls of plant roots are the first structures that come into contact with extracellular Cd. In *Lupinus albus*, these cell walls accumulate twice the amount of Cd as compared to that bound to intracellular thiol compounds. Within the stems and leaves, however, both quantities are approximately equal (Vazquez et al., 2006). In another study using *L. albus*, up to 88% of all Cd was found in the roots, with the amount of Cd bound to root cell walls being 37 times higher than that bound to leaf cell walls (Zornoza et al., 2002). Accumulation of Cd within the cell wall can reach even higher levels as was observed in shoots and roots of *Dittrichia viscosa* that accumulate almost all of the absorbed Cd within the cell wall fraction (Fernández et al., 2014). Additionally, in *A. thaliana* plants exposed to low Cd concentrations (1 μM), Cd was effectively retained within the cortical cell walls (Van Belleghem et al., 2007).

The capacity to retain Cd in the cell wall is determined by the amount of negatively charged functional moieties such as carboxyl, hydroxyl, and thiol groups (Krzesłowska, 2011). In a recent study on tomato suspension cells, Cd led to elevated levels of trace metal retention, which was explained by a 40% increase in cell wall biomass (Muschitz et al., 2015). Thickening of the cell wall also occurred in the vascular bundle cells of leaves and in xylem and phloem cells of roots in *Sorghum bicolor* exposed to 100 μM of CdCl_2_ (Jia et al., 2016). The reinforcement of the cell walls can be interpreted as a response of the plant to form a more effective barrier against the excessive amounts of Cd. Thereby, it has become clear that plants can actively modify their cell walls via a process termed cell wall remodeling.

### Primary Cell Wall Remodeling Related to Pectin

The best-studied cell wall modifications include changes in the distribution and abundance of esterified pectins (Douchiche et al., 2011). Upon Cd exposure, JIM5 epitopes – antibodies detecting demethylated forms of pectin – increased within the outer parts of the cell walls of *Linum usitatissimum* hypocotyls, reflecting the presence of low-methylated pectin, which is particularly able to bind Cd ions due to the presence of free carboxyl groups. However, within the inner part of the primary cell wall, the JIM7/JIM5 ratio [antibodies detecting highly methylated (JIM7) versus demethylated (JIM 5) forms of pectin] rose, which was hypothesized to have a repellent function in keeping the cytosolic Cd away from the fragile plasma membrane (Douchiche et al., 2007). This de-esterification was co-localized with an upregulation of PME activity complementary with an increased temporal activity of PODs. As will be discussed in section “Cadmium Stimulates POD Activity Leading to Increased Lignification,” these enzymes serve a primary role in the polymerization of lignin. Moreover, it was suggested that they might also function to crosslink HGAs once de-esterification by PME occurred (Paynel et al., 2009). Highly methylesterified pectin forms significantly decreased in a study by Muschitz et al. (2015) in which tomato cell suspension cultures were exposed to Cd. This was also combined with a decrease in PME activity. Additionally a reduction in pectin content was linked to a drop in gene expression of the *LeQUASIMODO1* gene (*LeQUA-1)*, which encodes a galacturonosyltransferase involved in pectin biosynthesis (Muschitz et al., 2015). In another study, the inhibitor cobtorin was used to disturb the parallel orientation of the cortical microtubili with cellulose microfibrils. It was hypothesized that cobtorin releases pectin from cellulose, forming gels that obstruct cellulose synthase from moving freely within the plasma membrane. Overexpression of PME suppressed this phenotype by making pectin bind more tightly to the cellulose microfibrils (Yoneda et al., 2010). However, the effect of PME on cell wall rigidness is not always clear. Besides crosslinking pectins through Ca bridges leading to an increased tightening of the cell wall, low-esterified pectins can also be a target of polygalacturonases, breaking these pectins down, thereby softening the cell wall (Levesque-Tremblay et al., 2015). These findings suggest that pectin modifications for sure play an important role in determining the cell wall stiffness, which eventually has major consequences for cellular growth and plant development, also under stress conditions such as Cd exposure. In two *Zea mays* hybrid plants with a different Cd tolerance, Cd exposure resulted in a drop in the cellulose content in both hybrids (Vatehova et al., 2016). This observation is in agreement with results obtained in *O. sativa* plants (Xiong et al., 2009). However, as the authors pointed out, these findings considering a cellulose decrease were not consistent with those obtained from dicot *L. usitatissimum* plants (Douchiche et al., 2007). Therefore, it was hypothesized that different defense responses are activated in monocot and dicot plants in response to Cd exposure (Parrotta et al., 2015; Vatehova et al., 2016).

To conclude, it is shown that the pectin content is increased within the cell wall after Cd exposure, and together with an enhanced PME activity, the production of low-methylesterified pectin is stimulated. Both modifications support an increased Cd retention capacity of the cell wall.

### Secondary Cell Wall Modifications Related to Lignin

Lignin is an important molecule protecting the protoplast by means of erecting a physical barrier against many biotic and abiotic stresses (Ederli et al., 2004). Lignification might make the cell wall less penetrable and therefore a more effective barrier against the entry of Cd, but it can also bind Cd itself (Parrotta et al., 2015). In plants exposed to Cd, lignification paradoxically protects the plant but can inhibit root growth if it occurs in the elongation zone, making it hard to assess the effectiveness of lignification in coping with Cd exposure (Schützendübel et al., 2001; Finger-Teixeira et al., 2010). However, comparing Cd-sensitive and -tolerant plants has often highlighted a difference in lignification that appeared key in explaining the mechanism of tolerance (Van De Mortel et al., 2006, 2008). In a comparison of mangrove trees and their sensitivity to Pb, Zn, and Cu, it was found that the more metal-tolerant species exhibited more lignification, and specifically more toward the root tips (Cheng et al., 2014). The hydrophobic lignin polymer is of particular interest when studying *in planta* effects of Cd toxicity. After all, its biosynthesis through oxidative polymerization is closely related to the redox state in the apoplast, which might be disturbed by metals such as Cd (Takahama and Oniki, 2000).

Lignification starts with the synthesis of monolignol subunits (section “The Structure of the Plant Cell Wall”), the building blocks of lignin. The generation of these soluble phenolics has frequently been observed in various plants exposed to Cd, often followed by increased POD activity and lignification (Schützendübel et al., 2001; Kováčik and Klejdus, 2008; Finger-Teixeira et al., 2010). Upstream, monolignols need to be synthesized via a complex network of enzymatic conversions (**Figure 2**). In Supplementary Table 1, the impact of Cd exposure on various key enzymes in monolignol biosynthesis at the transcript or activity level is summarized.

Phenylalanine ammonia lyase is one of the most frequently studied enzymes of the lignin biosynthesis pathway because (1) it is the first enzyme in the pathway, (2) it is highly stress-responsive, and (3) spectrophotometrical assays to measure its activity are available (Kováčik and Klejdus, 2012). Furthermore, there is evidence that this enzyme catalyzes the rate-limiting step in the phenylpropanoid pathway (Bate et al., 1994). Cadmium-induced increases in PAL activity have been observed in various plant species and organs (Supplementary Table 1), indicating an important role for the production of monolignols as a defense response (Hsu and Kao, 2005; Kováčik and Klejdus, 2008; Zheng et al., 2010; Yang et al., 2015). Glutathione is thought to be a key signaling molecule underlying the increased PAL activity, pointing toward the potential importance of PAL in mediating Cd tolerance (Wingate et al., 1988; DalCorso et al., 2010). In Cd-exposed *Matricaria chamomilla* plants, PAL activity increased (Kováčik et al., 2009a). In *Triticum aestivum* exposed to Cd and salicylic acid (SA), known to induce PAL, PAL activity increased by SA. This caused downstream lignin deposition that inhibited Cd uptake (Shakirova et al., 2016). Upon addition of SA to Cd-exposed *M. chamomilla*, however, PAL activity slightly increased in leaves and strongly decreased in roots, an effect associated with SA toxicity (Kováčik et al., 2009a). In *M. chamomilla* treated with the *in vivo* PAL inducer 2-aminoindane-2-phosphonic acid and exposed to Cd, PAL activity increased while the uptake of Cd decreased (Kováčik et al., 2011). Overall, these results indicate a role for increased PAL activity leading to Cd-induced lignification as an effective defense response.

## Cadmium-Induced Oxidative Stress and its Relation to the Cell Wall

**FIGURE 3:**
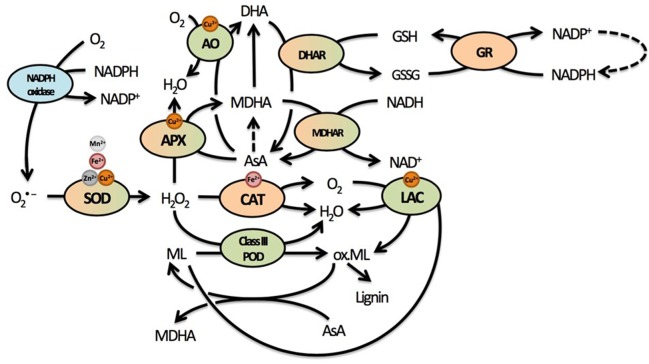
Summary of the most important elements of the cellular redox system related to cell wall responses in plants. The redox balance is maintained by an array of pro- and antioxidative metabolites and enzymes that form a complex network through regeneration and competition for substrates or supply thereof. Many of these enzymes use various micronutrients as important cofactors. Due to the oxidative nature of lignin polymerization, mainly through the actions of lignifying PODs, many enzymes could influence lignification in complex ways. Enzymes located exclusively in the apoplast are indicated in green, enzymes located exclusively in the protoplast in orange. Enzymes that have been found in both apoplast and protoplast are green and orange, while NADPH oxidase is a transmembrane protein usually found in the plasma membrane (indicated in blue). Dashed lines represent reactions requiring additional metabolites or enzymes that are not displayed here. Superoxide radicals are produced from O_2_ by NADPH oxidases. They are rapidly converted to the less reactive H_2_O_2,_ which is further reduced to H_2_O by several enzymes in different cellular compartments. Catalase is responsible for rapid detoxification of H_2_O_2_ in the peroxisome. In the cell wall, both APX and class III PODs compete to convert H_2_O_2_ into H_2_O. While APX uses AsA to facilitate this reaction and oxidize AsA to MDHA, class III PODs oxidize monolignols, thus facilitating lignification by the polymerization of radicalized monolignols. Another class of enzymes capable of oxidizing monolignols are LACs, thereby reducing O_2_ to H_2_O. Radicalized monolignols can, however, be scavenged by AsA, again stressing the important role of this antioxidant in lignification. In addition to APX, AO is present in the apoplast, competing for AsA to facilitate reduction of O_2_ to H_2_O while oxidizing AsA to DHA. The abundance of enzymes competing for H_2_O_2_ in the apoplast and using AsA highlight their importance in cell wall-related processes. Abbreviations: AO, ascorbate oxidase; APX, ascorbate peroxidase; AsA, ascorbate; CAT, catalase; DHA, dehydroascorbate; DHAR, dehydroascorbate reductase; GSH, glutathione (reduced); GSSG, glutathione (oxidized); GR, glutathione reductase; LAC, laccase; MDHA, monodehydroascorbate; MDHAR, monodehydroascorbate reductase; Ox. ML, oxidized monolignols; POD, peroxidase; SOD, superoxide dismutase.

Upon absorption, Cd causes damage through the indirect induction of oxidative stress (Cuypers et al., 2010; Petrov et al., 2015). Through its high affinity for thiol groups, Cd binds GSH and thereby depletes the reduced GSH pool (Jozefczak et al., 2015). Glutathione is an important antioxidant able to directly reduce ROS and regenerate AsA via the AsA-GSH cycle. Regeneration of AsA takes place in the cytosol, although various enzymes of the AsA-GSH cycle such as class I peroxidase APX, dehydroascorbate reductase (DHAR), and monodehydroascorbate reductase (MDHAR) are present in the apoplast as well (**Figure 3**) (Ferrer et al., 2001; Pignocchi and Foyer, 2003; Bielen et al., 2013). Ascorbate will be a focus point of the current review, because it is the most important apoplastic antioxidant based on its concentration. The presence of AsA and related enzymes in the apoplast additionally point to the importance of the cytosolic GSH pool in maintaining the apoplastic oxidative balance through the regeneration of AsA (Pignocchi and Foyer, 2003). In addition, this oxidative balance will strongly affect secondary cell wall formation, as lignification by class III PODs and LACs is driven by oxidative processes and influenced by competition for substrates (H_2_O_2_ and O_2_) to radicalize monolignols.

Ascorbate is not only an important antioxidant (Horemans et al., 2000), it also acts as a cofactor for numerous enzymes and a signaling molecule regulating pivotal cellular processes including cell division and expansion (de Pinto et al., 1999; Davey et al., 2000). In addition, it has been shown that plants with a decreased AsA concentration are more sensitive to environmental stresses (Koffler et al., 2015).

### Effect of Cd Exposure on AsA Concentrations

**FIGURE 4:**
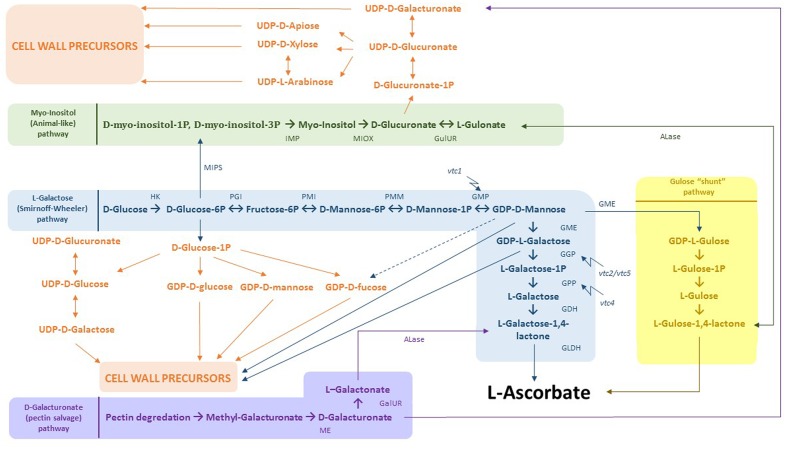
Integration of AsA biosynthesis and cell wall precursor metabolism. In blue: L-Galactose (Smirnoff-Wheeler) pathway. D-glucose derived from photosynthesis is converted to L-AsA by a 10 step process. Both D-glucose-6P and GDP-D-mannose can be used for the production of cell wall precursors. In yellow: gulose “shunt” pathway. GDP-D-mannose can additionally be converted to GDP-L-gulose by GME. Subsequently, GDP-L-gulose is dedicated to form the end product L-AsA. In green: myo-inositol (animal-like) pathway, where D-glucose-6P is processed to D-myo-inositol-1P/D-myo-inositol-6P. These products are then used to produce myo-inositol by myo-inositol monophosphatase. Next, MI can be consumed to create D-glucuronate as a direct cell wall precursor. In addition, D-glucuronate is the substrate to make L-gulonate, which flows directly into the gulose “shunt” pathway. In purple: D-galacturonate (pectin salvage) pathway. D-galacturonate derived from pectin degradation is converted to L-galactonate and further to L-galactose-1,4-lactone, the last intermediate within the L-galactose pathway. Abbreviations: HK, hexokinase; PGI, phosphoglucose isomerase; PMI, phosphomannose isomerase; PMM, phosphomannose mutase; GMP, GDP-mannose pyrophosphorylase, GME, GDP-3′,5′-epimerase; GGP, GDP-L-galactose phosphorylase; GPP, galactose-1-P phosphatase, GDH, galactose dehydrogenase; GLDH, galactono-1,4-lactono dehydrogenase; ME, methyl esterase; GalUR, D-galacturonate reductase; ALase, aldono-lactonase; MIPS, L-myo-inotsitol-1-phosphatase synthase; IMP, myo-inositol monophosphatase; MIOX, myo-inositol; GulUR, glucuronate reductase; *vtc1/2/4/5*, vitamin C 1/2/4/5 mutants.

Multiple studies have shown that Cd exposure affects AsA biosynthesis and its antioxidant properties in various plants (for a review, see Bielen et al., 2013). In *A. thaliana* plants grown and exposed to Cd in soil, three phases were described: (1) an alarm phase characterized by an initial drop of total leaf AsA concentrations within 12 h after exposure, (2) a resistance phase in which the total AsA concentrations began to rise again after 24 h, followed by (3) the exhaustion phase that led to a final AsA depletion between 7 to 14 days after exposure (Koffler et al., 2014). A similar response (resistance after short-term exposure) was observed in leaves of Cd-exposed *A. thaliana* plants grown hydroponically, where both GSH and AsA levels increased after 24 h (Keunen et al., 2013) as well as 72 h (Jozefczak et al., 2015). Root nodules of *Phaseolus vulgaris* plants exposed to 100 μM Cd for 4 days showed a significant decrease in the mRNA levels of genes encoding enzymes of the AsA biosynthesis pathway such as GDP-D-mannose-3′,5′-epimerase (GME), GDP-D-mannose phosphorylase, L-galactose dehydrogenase, and L-galactono-1,4-lactone dehydrogenase (**Figure 4**).

### Alterations to the Cell Wall Structure Mediated by the Oxidative Products of AsA

Through oxidation of AsA into monodehydroascorbate and dehydroascorbate, the cell wall structure can be altered via different mechanisms (reviewed in Davey et al., 2000; Bielen et al., 2013). Exogenously applied MDHA has been linked to cell wall expansion by solute uptake and increased activation of a H^+^ATPase in *Allium cepa* roots (Gonzalez-Reyes et al., 1995). In addition, DHA is able to interact with lysine and arginine side chains, preventing the binding of structural proteins to polysaccharides (Lin and Varner, 1991). Next, AsA is also the main starting point for oxalate production, thereby reducing free Ca availability within the apoplast. These Ca ions are also used in the crosslinking of different pectin molecules. Cell wall tightening could again be accomplished by oxalate oxidase, releasing Ca and H_2_O_2_ back in the apoplast (Smirnoff, 1996). Finally, lignification is also related to AsA concentrations and/or redox state, because AsA has the capacity to directly scavenge radicalized monolignols, preventing their oxidative polymerization into lignin (Takahama and Oniki, 1992; Otter and Polle, 1994). For example, a higher amount of AsA and/or a less oxidized pool correlates with decreased lignification as observed in *Picea abies* needles (Otter and Polle, 1994).

Ascorbate is also used by APXs, non-lignifying H_2_O_2_-detoxifying class I PODs that are also present in the same cellular compartments as class III PODs (De Gara, 2004). There, APX might compete with lignifying PODs for its substrate H_2_O_2,_ thereby inhibiting lignification. Additionally, ascorbate oxidase (AO) has been implicated in cell wall turnover, as this apoplastic enzyme competes with APX for AsA and subsequently oxidizes it to DHA while reducing O_2_ to H_2_O. A high amount of AO relative to APX might thus tip the balance in favor of more lignification by competing for AsA while leaving H_2_O_2_ free to be used as substrate for lignifying PODs. In roots of *P. abies* exposed to 50 μM Cd, a decreased APX activity was observed after 48 h, while guaiacol peroxidase (GPOD) activity and lignification increased (Otter and Polle, 1994). In conclusion, it becomes clear that AsA could alter cell wall structure in an indirect manner by its oxidative products, which all function within a complex redox network.

### The Biosynthesis Pathways of AsA and Cell Wall Components Are Strongly Entangled

In addition to the effects of MDHA and DHA, a closer look into the AsA biosynthesis pathway reveals several possible interactions between Cd and cell wall formation (**Figure 4**). The best-known AsA biosynthesis route constitutes the L-galactose or Smirnoff-Wheeler pathway (**Figure 4**, indicated in blue) (Wheeler et al., 1998). A total of 10 steps are completed, converting photosynthetically derived D-glucose into L-AsA (Linster and Clarke, 2008). Many studies have suggested that GDP-galactose phosphorylase is the key regulating enzyme, converting GDP-L-galactose into L-galactose-1P (step 7), thereby committing the pathway to its final product L-AsA (Bulley et al., 2009; Gilbert et al., 2009; Li et al., 2014). Both GME (step 6) and GDP-galactose phosphate phosphatase (GPP) (step 8) were shown to be responsive to abiotic stresses such as hypoxia, wounding and heat treatment (Li et al., 2013a,b). The GME enzyme is situated at an important crossing point joining AsA and cell wall component biosynthesis pathways. Both GDP-D-mannose and GDP-L-galactose are used as precursors by these last two pathways, resulting in competition for these products (Bulley and Laing, 2016). Furthermore, GDP-D-mannose can be converted to GDP-D-fucose, which is also a precursor of cell wall components such as xyloglucan polysaccharides. Additionally, GDP-L-galactose might be transported to the Golgi apparatus, where it is mainly incorporated into the pectin domain RGII by L-galactosyltransferases (Reuhs et al., 2004). Regarding regulation, GME was demonstrated to be inhibited by the end products L-galactose-1,4-lactone and L-AsA *in vitro* and could thereby function as a redox sensor controlling both antioxidant capacity and cell wall/glycoprotein synthesis (Wolucka and Van Montagu, 2003). High levels of GDP-D-fucose also contribute to the complex regulation of GME. Recently, a study of two *GME* genes in *Solanum lycopersicum* (*SlGME1 and SlGME2*) revealed tissue specificity and distinct functions for both genes. Only *SlGME2* RNAi lines showed an altered dimerization state of the pectin RGII (Mounet-Gilbert et al., 2016). This alteration was also seen in a study of Voxeur et al. (2011) using *S. lycopersicum* plants. There, transgenic *gme* lines showed a reduced capacity of RGII to perform crosslinking, thereby inhibiting the formation of a strong three-dimensional pectin network (Voxeur et al., 2011). In another study, *Slgme* knockdown lines showed increased mannose concentrations in the cell walls of both stems and fruits, leading to a galactoglucomannan-enriched cell wall. This observation was expected as the conversion of GDP-D-mannose to GDP-L-galactose was greatly reduced in these plants. In addition, they showed lower levels of methylated pectins and RGI galactan side chains. Taken together, these alterations in cell wall composition might result in different cell wall properties (Gilbert et al., 2009). To the best of our knowledge, very little is known on how Cd could influence the L-galactose pathway and by that affect the availability of precursors for cell wall construction.

Besides the Smirnoff-Wheeler pathway, three alternative pathways capable of producing AsA are proposed to exist: (1) the GDP-L-gulose pathway (**Figure 4**, indicated in yellow) (Wolucka and Van Montagu, 2003), (2) the myo-inositol (MI) (animal-like) pathway (**Figure 4**, indicated in green) (Lorence et al., 2004), and (3) the D-galacturonate (pectin salvage) pathway (**Figure 4**, indicated in purple) (Agius et al., 2003). To what extent these pathways contribute to the overall AsA production in plants is still unclear, since identification of these metabolic routes is mainly accomplished after ectopic expression of genes or exogenous substrate supplementation (Wheeler et al., 2015).

*Arabidopsis thaliana vtc2 vtc5* double mutants, deficient in the L-galactose pathway, also displayed a complete growth arrest after germination, indicating that the other pathways do not produce sufficient AsA to rescue the plant phenotype (Dowdle et al., 2007). Although these other pathways might contain additional regulation points for AsA production and cell wall biosynthesis upon Cd exposure, this is currently unexplored. Nevertheless, it is interesting to look at these pathways in more detail, since a strong connection between AsA and cell wall biosynthesis is suggested via these metabolic routes.

In addition to GDP-L-galactose, GME is also able to produce GDP-L-gulose (**Figure 4**, indicated in yellow) (Wolucka and Van Montagu, 2003). This product is further converted to L-gulose-1,4-lactone, finally resulting in L-AsA. In contrast to GDP-L-galactose, GDP-L-gulose is directly dedicated to form L-AsA (Wolucka and Van Montagu, 2003). To date, the overall contribution of this “gulose shunt” to AsA biosynthesis remains unclear (Bulley and Laing, 2016). Secondly, MI could be used as a possible precursor for AsA as evidenced by the identification of a MI oxygenase (MIOX) gene, located on chromosome 4 in *A. thaliana*, with the encoding enzyme converting MI into D-glucuronate (**Figure 4**, indicated in green) (Lorence et al., 2004). This metabolite could further be processed into L-gulonate and subsequently AsA. Plants overexpressing the *MIOX4* gene showed a two- to three-fold increase in AsA levels. However, this rise was not confirmed in a later study (Endres and Tenhaken, 2009). These authors concluded that the conversion of D-glucuronate into AsA only plays a minor role in determining total AsA content and that it is rather used to make UDP-D-glucuronate as a precursor for cell wall components. This reaction is catalyzed by a poorly characterized glucuronokinase, but is very likely to be a major controlling point between the two competing pathways. Thereby, this route provides an alternative to produce uronosyl and pentosyl residues, which can be incorporated into cell wall polysaccharides such as pectin and hemicellulose in addition to the already well-established oxidation of UDP-D-glucose to UDP-D-glucuronate (Loewus, 2006). An interesting finding that further links the Smirnoff-Wheeler pathway to MI production is the bifunctionality of the VTC4 enzyme (step 8) from the L-galactose pathway, which is able to hydrolyze both D-myo-inositol-3P and L-galactose-1P *in vitro* (**Figure 4**, indicated in green). Loss-of-function *vtc4* mutants additionally showed a lower supply of MI (Torabinejad et al., 2009). Finally, it is shown that upon fruit ripening, cell wall components can be remobilized through the hydrolysis of the pectin building blocks HGA and RGI, resulting in increased D-galacturonate levels (**Figure 4**, indicated in purple). Overexpression of a *GalUR Fragaria* × *ananassa* gene encoding a D-galacturonic acid reductase converting D-galacturonate into L-galactonate led to a two- to three-fold increase of AsA levels in *A. thaliana* (Agius et al., 2003). Transcripts of a pectinesterase and two polygalacturonases were also upregulated in an introgression tomato line (*Solanum pennellii* in a *S. lycopersicum* background) with higher AsA concentrations (Di Matteo et al., 2010). This pectin salvage route might be triggered by ethylene since several genes from the ethylene biosynthesis pathway appeared to be enhanced in fruits of the introgression line (Di Matteo et al., 2010).

### Antioxidative Defense or Cell Wall Retention upon Cd Exposure?

In the previous section, several links were revealed between the biosynthesis of AsA and cell wall components (**Figure 4**). Upon Cd exposure, this close entanglement between both pathways might allow plants to choose which of both defense strategies is employed. Upregulating AsA production creates more reducing power on the one hand, but also consumes some cell wall precursors and possibly counteracts cell wall construction on the other hand. To the best of our knowledge, very few reports have focused on how plants exposed to Cd alter these metabolic pathways. However, the *GME* gene has been proposed to integrate redox signals and adjust AsA biosynthesis (Wolucka and Van Montagu, 2003).

In *A. thaliana* plants, an immediate depletion of GSH occurs in roots after 2 h of Cd exposure. This decreased content of GSH is probably the result of an increased production of PCs, which form complexes with Cd once it has entered the cytosol. Nevertheless, other antioxidative mechanisms do not seem to be upregulated during this early response (Jozefczak et al., 2014). However, multiple antioxidant defense systems are activated after 24 h, including the production of a more reduced AsA pool. Therefore, after the early production of PCs, another defense against Cd exposure within the roots might be the construction of a more impermeable cell wall, in contrast to the investment in antioxidative metabolites. Such an early defense strategy might limit Cd uptake by the roots, thereby protecting the leaves from excess Cd as well.

Once Cd has entered the root system, it is partially translocated to the aboveground biomass. Because the plant does not benefit from reinforcing the cell wall of the leaves, one might hypothesize that a different defense strategy is being used in contrast to the roots. It is well known that Cd has some detrimental effects on photosynthesis by inhibiting enzymes from the Calvin cycle, interfering with stomatal conductance and reducing chlorophyll a/b content (Azevedo et al., 2005a; Ding et al., 2007; Dai et al., 2012; Koffler et al., 2014). Upon long-term Cd exposure, GSH and AsA are both redistributed to the chloroplasts, probably to detoxify photosynthetically derived ROS and thereby safeguard the plant’s energy production (Koffler et al., 2014). Also, it is observed that Cd concentrations within leaves stabilize after 24 h exposure in *A. thaliana* plants. This is accompanied by a delayed defense response when compared to the roots (Jozefczak et al., 2014). Therefore, it might be speculated that upregulating AsA biosynthesis is probably more preferred than boosting cell wall remodeling during Cd exposure in leaves as opposed to roots. However, since very little is known on how Cd influences AsA biosynthesis and how this is related to cell wall construction, further research within this area should be stimulated.

### Cadmium Stimulates POD Activity Leading to Increased Lignification

Lignification is closely tied to the oxidative state of the apoplast as lignifying enzymes radicalize monolignols, which can then polymerize into lignin (**Figure 2**) (Vanholme et al., 2010). Class III PODs are known to catalyze the polymerization of lignin using H_2_O_2_, which can be generated as reaction product of the detoxification of superoxide (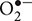) by SOD. These class III PODs are often referred to as lignifying PODs, but they are also involved in the polymerization of other cell wall components such as suberin (Cosio and Dunand, 2009). They are known to be secreted into and around the cell wall and their activities are routinely measured in spectrophotometric assays using guaiacol (GPOD) and syringaldazine (SPOD) substrates (Cesarino et al., 2013). The second class of enzymes involved in lignification are LACs. These are present in a variety of organisms and their involvement in lignification has been mostly deduced from their importance to degrade lignin in white rot fungi (Mayer and Staples, 2002). In spite of their hypothesized importance for lignification, the function of these Cu-containing enzymes remains largely unknown in plants and only a few have been functionally evaluated (Sato and Whetten, 2006; Bryan et al., 2016). It is known that they can oxidize and radicalize monolignols using O_2_ (Sterjiades et al., 1992). Furthermore, *A. thaliana* mutants without functional *LACCASE 4* and *17* genes showed a reduced lignin content, proving that this enzyme is required for normal lignification patterns (Berthet et al., 2011). One of the reasons for the current lack of knowledge is the inherent technical limitation when measuring LAC activity because of their shared substrate with many other PODs (Morales and Ros Barceló, 1997). Information on their role, if any, in lignification as an abiotic stress response remains especially scarce.

Cadmium exposure is known to disturb the oxidative balance and increase POD activity (Smeets et al., 2005, 2008, 2009). Increased POD activity can increase lignification, thereby inhibiting cell elongation and partially explaining the growth inhibition that is often a consequence of Cd toxicity (Schützendübel et al., 2001). However, this lignification might also be a defense response, immobilizing Cd in the secondary cell wall and/or preventing further absorption into the protoplast by mounting a physical barrier (Liu et al., 2004). Supplementary Table 2 provides an overview of studies that quantitatively determined the effects of Cd exposure on lignification, PODs or LACs activities as well as H_2_O_2_ as an important substrate for lignification.

In *Glycine max* roots, lignin content increased already after exposure to 1 μM Cd. In early stages (12-24 h), this correlated to an increased LAC activity, and a decreased POD activity. After 48 h, however, both LAC and POD activities were increased, indicating that LAC plays a role in the early stages of Cd toxicity while PODs become important at later stages (Yang et al., 2007). In another study on *G. max* roots of plants exposed to Cd for 24 h, increased POD activity was associated with a higher lignin content (Finger-Teixeira et al., 2010). In two studies on *M. chamomilla*, Cd exposure increased GPOD activity in both leaves and roots. The roots showed a slight increase in lignification after 7 days exposure, attesting to the high resistance of this plant to Cd as its growth was not inhibited (Kováčik and Klejdus, 2008; Kováčik et al., 2010). In comparison, Cu exposed roots showed bigger increases in lignification from the first day after exposure and experienced growth inhibition (Kováčik and Klejdus, 2008). Similar observations regarding GPOD activity were made in various organs of woody plants. In poplar stems and roots, 24 days exposure to 50 μM Cd led to an increase in GPOD activity and lignin content (Elobeid et al., 2012). Both GPOD activity and lignin content increased simultaneously after Cd exposure in *Pinus sylvestris* roots (Schützendübel et al., 2001).

As indicated in Supplementary Table 2, increases in both POD activity and lignification in response to Cd exposure have been observed in different organs of both herbaceous and woody plants. The question remains to what extent these responses contribute to a constitutive defense response to Cd exposure. Comparing Cd-tolerant and -sensitive cultivars can help to answer this question. In two *Oryza sativa* cultivars with a different Cd tolerance, the role of PODs was analyzed. Reduced growth and higher H_2_O_2_ accumulation were observed in Cd-sensitive varieties, whereas only an increased lignification was observed 96 h of exposure to 150 μM Cd in the Cd-tolerant variety. Overall, the POD activity was higher in response to Cd in the tolerant variety as compared to the sensitive one, and correlated also with an increase at the transcript level. Promoter analysis of both cultivars indicated that the Cd-tolerant variety had two additional conserved domains of a Cu-response element, potentially underlying its enhanced POD activity and lignification. Therefore, it was proposed that the synthesis of PODs and subsequent lignification in response to Cd resulted from the activation of this Cu-response element, assumed to be involved in Cd stimuli as well. The importance of Cu-response elements in the form of transcription factors and miRNAs for signal transduction in Cd exposure has recently been demonstrated in *A. thaliana.* Cadmium triggers a Cu-deficiency response that is regulated by these Cu-responsive elements (Gielen et al., 2016, 2017). Therefore, lignification could be a defense strategy accounting for the higher Cd tolerance of one of both varieties (Chang et al., 2012). Nevertheless, Cd exposure increased GPOD, SPOD, and LAC activities in roots of both Cd-tolerant and Cd-sensitive varieties of *Vicia sativa*. This was associated with increased lignin content, which was even higher in the Cd-sensitive variety. Activities of GPOD and SPOD but not LAC, were also higher in the sensitive variety, indicating a more prominent role for the former enzymes in lignification in response to Cd exposure. Furthermore, more lignin was deposited and more Cd accumulated in the roots of the Cd-sensitive as compared to the tolerant variety. This led Rui et al. (2016) to hypothesize that these cell wall responses were not a defense mechanism, but rather a consequence of Cd phytotoxicity. This difference with earlier findings in *Oryza sativa* might, however, be indicative of a different cell wall response to Cd exposure in monocots (such as *O. sativa*) and dicots (such as *V. sativa*). Additionally, Cd-tolerant varieties of *V. sativa* were shown to have a higher basal SOD activity that increased more in response to Cd as compared to a Cd-sensitive variety. For CAT, its basal activity was similar but displayed a much stronger increase in Cd-exposed tolerant versus sensitive varieties. This corresponded to POD activities and a stronger increase of lignification in response to Cd exposure in the Cd-sensitive variety. However, lignin deposition was correlated with an increase in Cd accumulation in the roots, whereas no difference in shoot accumulation was observed. The importance of the redox system rather than cell wall, as observed in this specific case, in the defense might be responsible for the differences observed between some monocots and dicots. In research comparing the effects of heavy metal toxicity on the monocot *Z. mays* and dicot *G. max*, similar amounts of lignification in response to Cd exposure were observed. However, dicots seemed to be more tolerant and show more elevated defense responses as well (Piršelová et al., 2011). In addition, SOD activity and H_2_O_2_ content were increased, the first being overall higher in the Cd-tolerant variety and the latter higher in the Cd-sensitive variety, similarly to the two *O. sativa* cultivars (Chang et al., 2012; Rui et al., 2016). The importance of redox enzymes in the lignification process in response to Cd toxicity was stressed as only a few differences in total phenolics but definite differences in lignin content were noted between the tolerant and sensitive *V. sativa* accessions (Rui et al., 2016). Of five *Medicago truncatula* ecotypes that were exposed to 100 μM Cd, four showed an increased GPOD activity, the highest increase being observed in the most tolerant accession. Histochemical analysis revealed accompanying strong lignification in the epidermal cells and xylem of all Cd-exposed roots, but not in the controls (Rahoui et al., 2016). Root growth was inhibited strongly in all accessions but less so in the tolerant ones. Lignification, specifically in the elongation zone, has been associated with root growth inhibition (Schützendübel et al., 2001; dos Santos et al., 2004). Nonetheless, the inhibition caused by lignification to limit Cd-uptake might be a minor trade-off compared to the damage suffered by the roots when Cd reaches the protoplast. Comparing accessions with a different Cd tolerance suggests that many different mechanisms seem to be at work in various plants. Indeed, even the PODs primarily responsible for lignification might vary between species, as indicated in *Citrus citumelo* rootstocks where a clear quantitative and histochemical correlation was found between Cd exposure, GPOD activity, H_2_O_2_ accumulation and lignification, but not between SPOD activity and either H_2_O_2_ accumulation or lignification (Podazza et al., 2012). Additionally, it should be kept in mind that class III PODs, albeit vital, are not the only link in a complex chain of redox enzymes that can eventually lead to lignification.

### Lignifying PODs Participate within a Complex Redox Network in Plants with H_2_O_2_ at the Center

Lignifying PODs tie into a complex network of ROS, pro- and antioxidative metabolites and enzymes (**Figure 3**). On the one hand, the polymerization of monolignols requires their radicalization. On the other hand, lignifying PODs and LACs use H_2_O_2_ and O_2_ as substrates, respectively. A summary of the actions performed by the most important antioxidative enzymes discussed within this review and their substrates is presented in **Figure 3**. In spite of the observed differential responses of these enzymes to Cd in various plants with a different Cd tolerance, only limited research has been devoted to their role in the lignification response (Schützendübel et al., 2001; Podazza et al., 2012; Rahoui et al., 2014, 2016; Rui et al., 2016).

The role of AsA as both a direct scavenger and substrate for competing PODs (APX) was discussed in section “Alterations to the Cell Wall Structure Mediated by the Oxidative Products of AsA.” However, also H_2_O_2_ supply might control the extent of lignification. Superoxide dismutases use several metals as cofactors catalyzing the electron transfer required to reduce 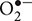 to H_2_O_2_. Examples are CuZnSOD, FeSOD, and MnSOD (Alscher et al., 2002). The existence of extracellular CuZnSODs has been confirmed in various plants and would suggest they are the key isoform involved in the Cd-induced lignification process (Streller and Wingsle, 1994; Ogawa et al., 1996; Alscher et al., 2002; Kasai et al., 2006). The homeostasis of Cu and Zn can be disturbed by Cd and it was shown that Cd exposure strongly reduced CuZnSOD activity in *P. sativum* plants (Sandalio et al., 2001; Rodríguez-Serrano et al., 2009). Similarly, expression of *COPPER/ZINC SUPEROXIDE DISMUTASE 1* and *2* was suppressed by Cd exposure in *A. thaliana* (Cuypers et al., 2011). Multiple results point toward a role for CuZnSOD in lignification, supplying H_2_O_2_ as a substrate for lignifying PODs. In *P. sylvestris* roots exposed to 50 μM Cd, SOD activities showed a fast but transient increase after 12 h before increases in POD activity and lignification were noted (at 48 h). However, changes in SOD activity could not be attributed to cytosolic CuZnSOD at the gene expression level, nor could the total SOD activity be explained by increases in FeSOD and MnSOD activity, suggesting the involvement of extracellular SODs (Schützendübel et al., 2001). Through histochemical analyses of the cellular localization of SOD, 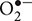 generation and lignification in *Spinacia oleracea* hypocotyls, the presence of CuZnSOD in the apoplast correlated to sites of lignification (Ogawa et al., 1996). Further research also confirmed the upstream role of NADPH oxidase in lignification by supplying 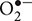 as a substrate to CuZnSOD (Ogawa et al., 1997). In addition, NADPH oxidase could be a key player in mitigating the cell wall responses to Cd exposure. Indeed, NADPH oxidase is proposed to significantly contribute to the ROS-generating cascade in response to Cd exposure (Remans et al., 2010). In addition, NADPH oxidases are located in close proximity to the apoplast because they are transmembrane proteins that shuttle electrons from donors in the cytosol to the extracellular space (Sagi and Fluhr, 2006; Jiménez-Quesada et al., 2016).

In contrast to SOD, CAT, and APX activities have been observed to initially decrease but strongly increase after 24 h of Cd exposure. This response was attributed to the initial depletion of GSH by Cd and subsequent increased GSH synthesis by H_2_O_2_ as signal molecule (Schützendübel et al., 2001). In Cd-sensitive (*Citrus sinensis* × *Poncirus trifoliata* and *Citrus volkameriana*) versus -tolerant (*Citrus paradise* × *Poncirus trifoliata* and *Citrus limonia*) citrus rootstocks, different responses related to SOD and CAT were observed that could not be directly correlated to one tolerance mechanism (Podazza et al., 2016).

A recent study using transgenic *A. thaliana* plants overexpressing SOD and APX under control conditions indicated positive correlations between the activity of APX and SOD and lignification (Shafi et al., 2015). Overexpression of either only SOD or both SOD and APX was associated with increased lignification at sites of CuZnSOD distribution, again pointing to the importance of this specific SOD isoform in lignification. Transcriptome analysis of these transformants indicated that H_2_O_2_ accumulating through SOD overexpression activates a large amount of lignin biosynthesis genes. Furthermore, transgenic lines exhibited similar H_2_O_2_ levels to wild-type (WT) plants under control conditions, but higher levels under salt stress. The accession overexpressing SOD accumulated the highest levels of H_2_O_2_, followed by the accession overexpressing both SOD and APX and finally the accession overexpressing APX only. Furthermore, transcription factors regulating lignin biosynthesis were identified and their activation by SOD overexpression suggests a role for H_2_O_2_ as signaling molecule leading to lignification rather than only acting as a substrate for lignifying enzymes (Shafi et al., 2015). Production and scavenging of H_2_O_2_ by SOD and APX, respectively, can thus be part of a control mechanism for lignification, also upon Cd exposure in plants.

**FIGURE 5:**
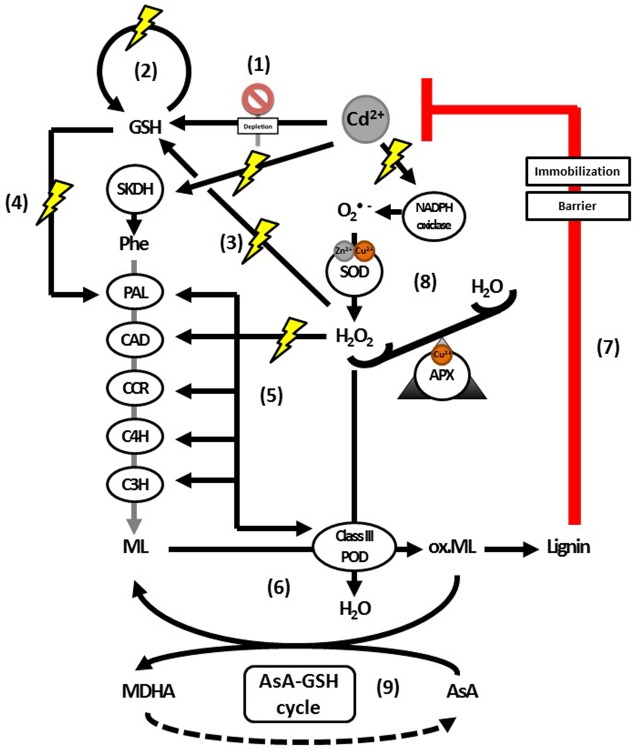
Proposed model for the actions of H_2_O_2_ as a signaling molecule and substrate for lignification activated upon Cd exposure. Glutathione depletion is an immediate effect of Cd exposure (1). This depletion is followed by an upregulation of GSH biosynthesis (2). Hydrogen peroxide has been implied as signaling molecule regulating this upregulation (3). Glutathione is important for H_2_O_2_ homeostasis and a key component in the transcriptional activation of PAL (4). Enzymes further downstream in the lignin biosynthesis pathway were shown to be activated by H_2_O_2_ (5), although it is currently not known whether GSH is able to transcriptionally activate them as well. Furthermore, H_2_O_2_ leads to increased biosynthesis of and is a key substrate for lignifying PODs (6), the last step in lignification as an effective defense response to Cd (7). The levels of H_2_O_2_ required to activate monolignol biosynthesis transcription (5) or provide sufficient substrate for monolignol oxidation by class III PODs (6), are maintained by APX (8). In addition to being an essential substrate of APX, AsA is able to scavenge radicalized monolignols (9), with a correlation between lower or more oxidized pools of AsA and decreased lignification. Abbreviations: AsA, ascorbate; APX, ascorbate peroxidase; C3H, coumarate 3-hydroxylase; C4H, cinnamate 4-hydroxylase; CAD, cinnamyl alcohol dehydrogenase; CCR, cinnamoyl-CoA reductase; GSH, glutathione (reduced); MDHA, monodehydroascorbate; ox. ML, radicalized monolignols; PAL, phenylalanine ammonia lyase; Phe, phenylalanine; POD, peroxidases; SKDH, shikimate dehydrogenase; SOD, superoxide dismutase.

Peroxidases require H_2_O_2_ to polymerize monolignol subunits into lignin. The importance of H_2_O_2_ in lignification has been indicated by increases in H_2_O_2_ correlating with increased GPOD and SPOD activity and lignification after Cd exposure (Supplementary Table 2). Lignification could therefore be a common plant defense pathway upon the overwhelming of the antioxidative defense capacity such as the GSH pool. In *A. thaliana* plants suffering from salt stress, a model was proposed where 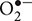 radicals, generated by NADPH oxidase, are converted into H_2_O_2_ by SOD (Shafi et al., 2015). Subsequently, H_2_O_2_ levels are controlled by APX and used as a signal to transcriptionally activate lignification (Shafi et al., 2015). It has been suggested before that APX has a role in the fine-tuning of H_2_O_2_ levels, which were shown to affect cell wall synthesis both directly and through upregulation of lignin biosynthesis genes and associated transcription factors (Mittler, 2002; Shafi et al., 2015). Thus, in addition to its role as a substrate, H_2_O_2_ appears to have a signaling function to stimulate lignification. Similar models could exist for Cd stress, known to indirectly increase H_2_O_2_ levels as well (Supplementary Table 2) (Finger-Teixeira et al., 2010; Chang et al., 2012; Kováčik and Klejdus, 2012; Cuypers et al., 2016; Rui et al., 2016). For example, NADPH oxidase contributes to the Cd-induced ROS cascade as indicated in **Figure 5** (Remans et al., 2010). In addition, lignin might not be the only secondary cell wall component of which the deposition is controlled by H_2_O_2_ signaling. In *Gossypium hirsutum* plants with secondary cell walls that are almost entirely made up of cellulose, inhibition of H_2_O_2_ formation interfered with cellulose deposition in the secondary cell wall and conversely, exposure to H_2_O_2_ promoted this process (Potikha et al., 1999).

The activation of monolignol biosynthesis enzymes at the transcript and activity level as well as the accumulation of soluble phenolics observed upon Cd exposure, suggest that lignification is more than the mere consequence of a disturbed redox balance in the apoplast affecting oxidative polymerization. The metabolism of GSH is closely linked to H_2_O_2_ homeostasis through its direct ROS scavenging capacity and its role as a substrate for glutathione reductase, contributing to AsA regeneration within the AsA-GSH cycle (Quan et al., 2008; Bielen et al., 2013). Conversely, H_2_O_2_ is known to increase GSH levels, suggesting a signaling role for H_2_O_2_ in mediating this response (Queval et al., 2009). While expression levels of genes encoding GSH biosynthesis enzymes were not increased after H_2_O_2_ application (Queval et al., 2009), they did increase after Cd exposure in *A. thaliana* plants (Jozefczak et al., 2014). In addition to its role as Cd-chelating agent, GSH is thought to be a key player in the stimulation of PAL, the first enzyme within the lignin biosynthesis pathway (**Figure 2**) (Rüegsegger et al., 1990; Edwards et al., 1991; Jozefczak et al., 2015). Glutathione was shown to activate PAL transcription and activity in *P. vulgaris* suspension cells (Wingate et al., 1988; Edwards et al., 1991). No such increase was observed in *Medicago sativa* suspension cells, which could be attributed to differences in uptake and metabolism of exogenously applied GSH rather than different signaling properties (Edwards et al., 1991). Undoubtedly, more research is needed on the relationship between PAL and GSH in Cd-exposed plants.

Further downstream in lignin biosynthesis, *CINNAMYL ALCOHOL DEHYDROGENASE* promoter expression was increased by exogenous H_2_O_2_ treatment in *Ipomoea batatas* plants (Kim et al., 2010). In *Saccharum* spp., addition of low H_2_O_2_ concentrations resulted in a transcriptional induction of *PAL*, *C4H*, *C3′H*, and *CCR* genes. Higher concentrations, however, repressed the phenylpropanoid pathway enzymes at the level of transcription, pointing to the importance of well-regulated levels of H_2_O_2_ for signaling (Arencibia et al., 2012). Moreover, H_2_O_2_ can also act as a signaling molecule to increase the amount of lignifying PODs. In transgenic *Solanum tuberosum* plants with elevated H_2_O_2_ concentrations, more extracellular PODs accumulated as compared to WT plants, suggesting that the signaling role of H_2_O_2_ stimulating lignin biosynthesis extends to the very last step of lignification (Wu et al., 1997). Hydrogen peroxide might thus serve a dual role in Cd-induced cell wall responses: (1) stimulating the biosynthesis of soluble phenolics via transcriptional activation of the lignin biosynthesis pathway and (2) acting as substrate for lignifying PODs to drive oxidative polymerization of monolignols to lignin.

In addition to H_2_O_2_, other signaling components might mediate cell wall-related responses upon Cd stress. For example, several studies have shown that Cd exposure stimulates the biosynthesis of ethylene and signaling by this phytohormone (reviewed by Keunen et al., 2016). Ethylene is well known to inhibit cell expansion in roots and shoots, mainly by influencing the transport and synthesis of auxin (Vandenbussche et al., 2012; Van de Poel et al., 2015). Furthermore, it was shown to increase the activities of cell wall hydrolases such as PME during ethylene-induced ripening of *Musa acuminata* fruit (Lohani et al., 2004). In P deficient *O. sativa* plants, Zhu et al. (2016) have shown that addition of the ethylene precursor 1-aminocyclopropane-1-carboxylic acid significantly increased the pectin content of the cell wall, ultimately contributing to root P remobilization. Furthermore, it was recently demonstrated that nitric oxide (NO^•^) acts upstream of ethylene within this process (Zhu et al., 2017). Reciprocally, pectic material itself might be an endogenous trigger of ethylene production and ripening (Tong and Gross, 1990). Also, inhibition of cellulose synthesis was shown to activate ethylene-dependent stress responses in *A. thaliana* (Ellis et al., 2002). These results point toward a reciprocal interaction between the cell wall and ethylene signaling in plants, which deserves further attention under stress conditions such as Cd exposure. Indeed, it has been suggested that cell wall remodeling and ethylene signaling are mediating salt acclimation in *A. thaliana* plants, at least at the transcriptional level (Shen et al., 2014). Ethylene is able to affect ROS producing as well as scavenging enzymes and metabolites (reviewed by Keunen et al., 2016). For example, it is an important upstream regulator of 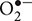-producing NADPH oxidases (Chae and Lee, 2001). In addition, ethylene insensitive *ein2-5* mutant plants produced less H_2_O_2_ as compared to their WT counterparts during mercury exposure (Montero-Palmero et al., 2014). By affecting the production of ROS or reactive nitrogen species such as NO^•^ upon Cd exposure, ethylene might contribute to or even underlie cell wall-related responses as discussed before.

## Conclusion and Future Perspectives

The cell wall is the most important structure in retaining Cd, thereby limiting the extent of damage in the protoplast. A diverse array of molecules within the cell wall shows an adaptive response to Cd exposure in plants. Pectin was highlighted as a key molecule to sequester bivalent ions in the primary cell wall. Additionally, AsA modulates cell wall responses by acting as the most important apoplastic antioxidant and sharing several links with cell wall component biosynthesis. In the secondary cell wall, lignification constitutes the main defense against Cd toxicity, with a central role for H_2_O_2_ as signaling molecule and substrate. Cell wall responses to Cd exposure are species-dependent, with potential differences between monocots and dicots. Nonetheless, the majority of studies demonstrate the activation of specific lignin biosynthesis enzymes to finally immobilize Cd. Future research could be aimed to further explore the role of H_2_O_2_, AsA, GSH, and other signaling compounds such as ethylene in cell wall-related responses to Cd stress in plants.

## Author Contributions

All authors participated in the conception of the topic. CL, MH, EK, and AC wrote the manuscript. CL and MH designed the figures, CL the Supplementary Tables. All authors read and approved the final manuscript after critically revising it for important intellectual content.

## Conflict of Interest Statement

The authors declare that the research was conducted in the absence of any commercial or financial relationships that could be construed as a potential conflict of interest.
