# Corrigendum: Reciprocal Interactions between Cadmium-Induced Cell Wall Responses and Oxidative Stress in Plants

**DOI:** 10.3389/fpls.2018.00391

**Published:** 2018-03-23

**Authors:** Christophe Loix, Michiel Huybrechts, Jaco Vangronsveld, Marijke Gielen, Els Keunen, Ann Cuypers

**Affiliations:** Environmental Biology, Centre for Environmental Sciences, Hasselt University, Diepenbeek, Belgium

**Keywords:** cadmium, oxidative stress, cell wall, pectin, lignin, ascorbate, hydrogen peroxide

In the original article, there was a mistake in the legend for Figure 2 as published. An incorrect version was submitted. The correct legend appears below.

**Figure 2:**
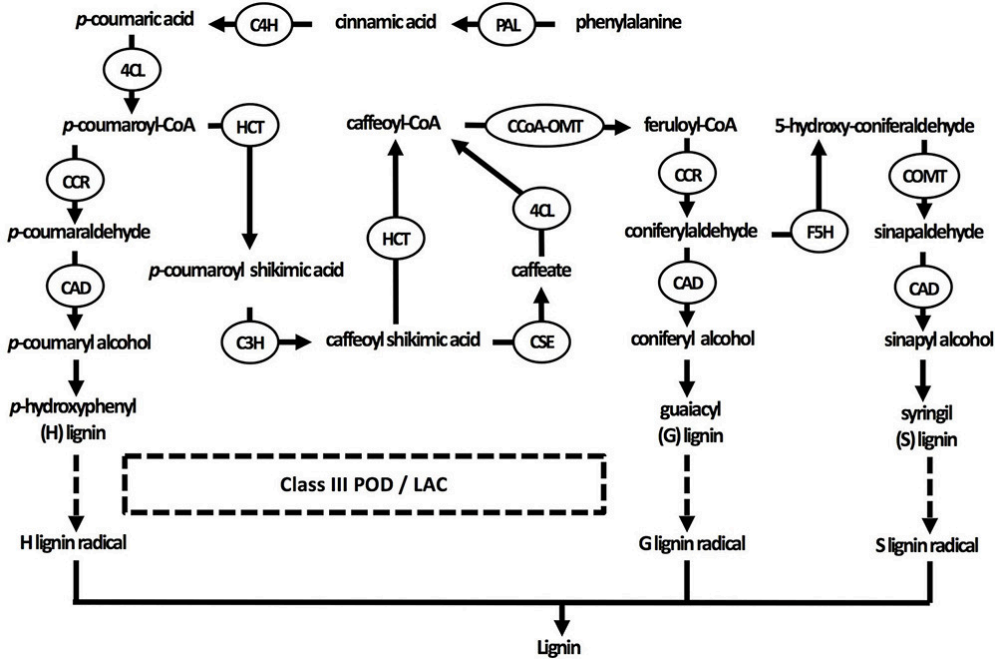
Current understanding of the lignin biosynthesis pathway in plants. Three specific monolignol subunits are synthesized from phenylalanine by enzymes that are sometimes able to catalyze different reactions in the pathways of all three monolignols. Monolignols are subsequently transported to the cell wall, where they are radicalized by class III peroxidases and laccases in the final step of lignification (indicated by dashed arrows). 4CL, 4-coumarate-CoA ligase; C3H, coumarate 3-hydroxylase; C4H, cinnamate 4-hydroxylase; CAD, cinnamyl alcohol dehydrogenase; CCoA-OMT, caffeoyl-CoA O-methyltransferase; CCR, cinnamoyl-CoA reductase; COMT, caffeic acid O-methyltransferase; F5H, ferulate 5-hydroxylase; HCT, *p*-hydroxycinnamoyl-CoA: quinate/shikimate *p*-hydroxycinnamoyltransferase; LAC, laccase; PAL, phenylalanine ammonia-lyase; POD, peroxidase.

In the original article, there was a mistake in Figure 2 as published. An incorrect version was submitted. The correct Figure 2 appears below.

In the original article, there was an error. An enzyme name was wrong.

A correction has been made to Cadmium induces cell wall remodeling, The Structure of the Plant Cell Wall, page 5:

After conversion of *p-*coumaric acid to *p-*coumaroyl-CoA by 4-coumarate-CoA ligase, *p-*coumaroyl-CoA can follow one of three pathways that are specific but still share common enzymes, leading to the formation of three possible alcohols.

The authors apologize for these errors and state that this does not change the scientific conclusions of the article in any way.

The original article has been updated.

## Conflict of interest statement

The authors declare that the research was conducted in the absence of any commercial or financial relationships that could be construed as a potential conflict of interest.

